# An In Vivo Drug Screen Reveals That Sirtuin 2 Activity Promotes Spinal Cord Neurogenesis in Developing Zebrafish

**DOI:** 10.3390/biom15101359

**Published:** 2025-09-24

**Authors:** Laura González-Llera, Álvaro J. Arana, Laura Sánchez, Antón Barreiro-Iglesias

**Affiliations:** 1 Departamento de Bioloxía Funcional, Facultade de Bioloxía, Universidade de Santiago de Compostela, 15782 Santiago de Compostela, Spain; laura.gonzalez.llera@rai.usc.es; 2 Aquatic One Health Research Center (ARCUS), Universidade de Santiago de Compostela, 15782 Santiago de Compostela, Spain; 3 Department of Zoology, Genetics and Physical Anthropology, Faculty of Veterinary Science, Universidade de Santiago de Compostela, 27002 Lugo, Spain; alvaro.arana@usc.es (Á.J.A.); lauraelena.sanchez@usc.es (L.S.); 4 Preclinical Animal Models Group, Health Research Institute of Santiago de Compostela (IDIS), 15706 Santiago de Compostela, Spain

**Keywords:** spinal cord, drug screen, neurogenesis, sirtuin 2, serotonin, zebrafish, mitosis, acetylated alpha-tubulin

## Abstract

Given the central role of neurogenesis in building a functional nervous system, we recently developed a zebrafish-based drug-screening protocol to uncover molecules and signalling pathways regulating spinal cord neurogenesis. In this study, we have expanded this drug screen and discovered a previously unknown role of deacetylase sirtuin 2 (SIRT2) in promoting the generation of serotonergic interneurons in the spinal cord. Treatments with specific SIRT2 inhibitors reduced the generation of serotonergic neurons in the spinal cord, which led to locomotor deficits. Our data suggest that SIRT2 regulates mitotic activity in progenitor cells to promote the generation of serotonergic neurons in developing animals. Together, our results uncover SIRT2 as a key regulator of spinal cord neurogenesis and position it as a promising target for strategies aimed at neural repair in spinal cord disorders.

## 1. Introduction

The neural circuits located in the spinal cord play a key role in modulating and generating locomotor movements in vertebrates (see [1]); therefore, finding the molecules and signalling pathways that regulate spinal cord neurogenesis during early development is of significant interest. This knowledge could help in identifying new molecular targets to enhance neuronal regeneration or survival following traumatic spinal cord injuries or in neurodegenerative disorders affecting the spinal cord.

The transparency and rapid development of zebrafish embryos and larvae make them a valuable animal model for the study of the molecular pathways regulating neurogenesis in the spinal cord (e.g., [2,3,4,5,6,7,8]). Moreover, their small size and ability to absorb drugs through the skin and into the central nervous system (CNS) also offer an excellent model system to perform high-throughput and high-content phenotypic drug screening in vivo. We have recently developed a drug-screening protocol to identify small molecules and signalling pathways regulating neurogenesis in the developing zebrafish spinal cord [8]. For this, we used as a cellular model the population of serotonergic interneurons of the ventral spinal cord [8]. This neuronal population offers a good model for drug screens because serotonergic neurons originate from lateral floor plate progenitor cells [6] between 48 and 60 h post-fertilisation (hpf) [9], a period that follows the main embryonic morphogenic events. Thus, drugs can be applied to 2-day post-fertilisation (dpf) animals, ensuring that any observed effects are more likely to be cell-specific rather than due to interference with major morphogenic processes. Using this protocol, we initially screened 160 small molecules of the LOPAC^®^ Library (Sigma), which led us to discover a role for prostaglandin D_2_ in promoting neurogenesis in the ventral spinal cord [8]. Here, our first aim was to expand the LOPAC^®^ Library drug screen by testing 80 additional compounds to identify the most promising candidate for further experimentation. As indicated below, this drug screen revealed a possible role for the deacetylase sirtuin 2 (SIRT2; also known as silent information regulation protein) in promoting neurogenesis in the spinal cord (see below). Therefore, the second aim of our study was to analyse the role of this deacetylase in regulating spinal cord neurogenesis in zebrafish.

SIRT2 is a member of the sirtuin family of proteins, which includes seven mammalian members. Sirtuins are protein deacetylases that share highly conserved central NAD+ binding and catalytic domains [10]. SIRT2 is mainly found in the cytoplasm but can also be found in the cell nucleus [10]. SIRT2 can deacetylate many substrates like histones 3 and 4 or alpha-tubulin (for a review see [11]). Interestingly, SIRT2 is highly expressed in the mammalian CNS, particularly in the cortex, striatum, hippocampus, and spinal cord, suggesting that it may play important roles in these CNS regions [11,12]. Regarding its role in neurogenic processes, SIRT2 has been implicated in the differentiation of dopaminergic neurons in the substantia nigra of mice [13], and recent work has revealed a link between decreased levels of SIRT2 in animal models of depression and reduced hippocampal neurogenesis [14,15]. However, there is no prior knowledge on the role of SIRT2 in the regulation of spinal cord neurogenesis in developing animals. In this paper, we provide compelling pharmacological data showing that Sirt2 activity in the developing zebrafish spinal cord promotes the generation of serotonergic neurons by regulating the mitotic activity of progenitor cells.

## 2. Materials and Methods

### 2.1. Animals

Wild-type zebrafish were kept and raised under standard conditions [16] in the fish facilities of the Department of Genetics of the University of Santiago de Compostela (codes of the facility: AE-LU-003, ES270280346401). All experiments were approved by the Bioethics Committee of the University of Santiago de Compostela and the Xunta de Galicia (project license no.: 01/20/LU-003; approved on 1 March 2020) and were carried out in accordance with the EU Directive 2010/63/EU for animal experiments. For experimental analyses, we used wild-type larvae (between 2 and 4 dpf). Embryos were collected from the breeding tanks and were divided into Petri dishes with 30 mL of fish water (reverse-osmosis-purified water) at a density of 80 to 100 embryos per dish until they were 2 dpf (when they were used for drug treatments, see below), although no formal randomisation method was used. The specific number of animals used for each experiment is indicated in the figures or in the Supplementary Files.

### 2.2. Drug Screen

We carried out an unbiased drug screen of the LOPAC^®^ 1280—Small-Scale Library (Sigma, St. Louis, MO, USA; Cat# LO4200-1EA; International Version). We screened drugs (at 10 µM) from rack 1 of the library (80 drugs in total; stocks at 10 mM in DMSO) following the 2-step in vivo drug-screening protocol developed by González-Llera et al. [8]. Zebrafish larvae were incubated with each drug from 2 dpf to 4 dpf in groups of 5 animals per well in 24-well plates. Control animals were treated with DMSO.

### 2.3. Treatments with Specific Drugs in Whole Larvae

Specific drugs (SirReal2: Sigma, Cat# SML1514; AGK2: Sigma, Cat# A8231) were diluted in DMSO at a stock concentration of 10 mM. For the treatments, 2 µL of the stock solution were diluted in 2 mL of fish water (reverse-osmosis-purified water; working dilution of 10 µM). Larvae were incubated with each drug from 2 dpf to 3 or 4 dpf in groups of 5 animals per well in 24-well plates. Control animals were treated with 2 µL of DMSO in 2 mL of fish water.

### 2.4. Behavioural Analyses

For behavioural (swimming) analyses, 4 dpf larvae were transferred to 24-well plates (1 animal per well). Before measuring the locomotor activity, the larvae were left in clean water without SirReal2 or DMSO (control group) for 1 h. Locomotor activity was assessed in a Zebrabox (Viewpoint Life Sciences; Civrieux, France) using a dark–light test consisting of three alternating light and dark cycles, each lasting 6 min (36 min in total). Tracking data were obtained using Zebralab (Viewpoint Life Sciences), which provided the individual parameters of total swimming distance, number of locomotor events, and duration of activity. The scale factors provided by the system were x scale = 1.14094, y scale = 1.14094, and scale unit = mm, allowing for the spatial data to be expressed in millimetres.

### 2.5. Whole-Mount Anti-Serotonin Immunofluorescence

After the drug treatments, 4 dpf larvae were euthanised by tricaine methanesulfonate (Sigma) overdose and then fixed with 4% paraformaldehyde (PFA) in phosphate-buffered saline (PBS; pH 7.4) for 2 h at 4 °C. After washing in PBS, 4 dpf larvae were incubated in Proteinase K from *Tritirachium album* (Sigma; Cat#: P4850; ≥800 U/mL; 1 µL per mL of PBS) for 35 min at room temperature and then in glycine [50 mM in PBS with 0.2% Triton X-100 (PBST)] for 10 min at room temperature. Then, the larvae were incubated with a rabbit anti-serotonin antibody (dilution 1:2500; Immunostar, Hudson, WI, USA; Cat#: 20080; RRID: AB_572263) overnight at 4 °C. Then, they were rinsed in PBST and incubated overnight at 4 °C with a Cy3-conjugated goat anti-rabbit antibody (dilution 1:500; Jackson ImmunoResearch, West Grove, PA, USA; Cat#: 111-165-144; RRID: AB_2338006). Antibodies were diluted in PBST with 1% DMSO, 1% normal goat serum, and 1% bovine serum albumin. Larvae were mounted with 70% glycerol in PBS. Control and treated animals were always processed in parallel, and the same antibody solutions were used for both the control and treated animals in each experiment. Controls omitting the primary antibody led to no staining at all.

### 2.6. Cryostat Sections

The 2 or 3 dpf larvae were euthanised by tricaine methanesulfonate (Sigma) overdose and then fixed with 4% PFA in PBS (pH 7.4) for 2 h at 4 °C. After rinsing in PBS, larvae were cryoprotected overnight with 30% sucrose in PBS, embedded in Neg-50^TM^ (Thermo Scientific, Waltham, MA, USA), and frozen with liquid-nitrogen-cooled isopentane. Transverse sections of the body, starting from the caudal fin (16 μm in thickness) and moving rostrally, were obtained on a cryostat and mounted on Superfrost Plus slides (Epredia, Portsmouth, New Hampshire, USA).

### 2.7. TUNEL Labelling on Cryostat Sections

TUNEL staining was performed according to the manufacturer’s protocol with minor modifications (In Situ Cell Death Detection Kit, TMR red; catalogue number 12156792910; Roche, Basel, Switzerland). Briefly, the sections were incubated in methanol for 15 min at −20 °C to permeabilise the lipid membranes, followed by brief washes in PBS, and another incubation in 0.01 M citrate buffer pH 6.0 for 30 min at 70 °C. After several washes in PBS, sections were incubated in the TUNEL reaction mix, containing the labelling solution (TMR-red–labelled nucleotides) and the enzyme solution (terminal deoxynucleotidyl transferase), for 90 min at 37 °C. Slides were washed in PBS and distilled water, allowed to dry for 30 min at 37 °C, and mounted with MOWIOL^®^ 4-88 (Calbiochem, San Diego, CA, USA). Sections from the control and treated animals were processed in parallel, and the same TUNEL labelling solution was used for sections from control and treated animals. Negative controls were obtained by incubating sections only with the labelling solution (without terminal deoxynucleotidyl transferase). Positive controls were previously generated [8] by incubating some sections from 3 dpf animals with recombinant DNAse I (400 U/mL; Roche) for 20 min at room temperature before the incubation with the reaction mix.

### 2.8. Immunofluorescence on Cryostat Sections

Sections were first treated with 0.01 M citrate buffer, pH 6.0, for 30 min at 90 °C for heat-induced epitope retrieval, allowed to cool for 10 min at room temperature in distilled water, and then rinsed in 0.05 M Tris-buffered saline (TBS), pH 7.4, for 20 min. Then, the sections were incubated overnight at room temperature with a rabbit polyclonal anti-pH3 antibody (1:500; Sigma, Cat#: H0412; RRID: AB_477043), a mouse monoclonal anti-acetylated alpha-tubulin antibody (1:500; Sigma, Cat#: T6793; RRID: AB_477585), or a rabbit polyclonal anti-SIRT2 antibody (1:100; Sigma, Cat#: S8477; RRID: AB_1079981). Sections were then rinsed 3 times in TBS for 15 min each and incubated for 1 h at room temperature with a Cy3-conjugated goat anti-rabbit IgG antibody (1:500; Jackson ImmunoResearch, Cat#: 111-165-144; RRID: AB_2338006) or an FITC-conjugated goat anti-mouse antibody (1:200; Invitrogen, Carlsbad, CA, USA, Cat# F2761; RRID: AB_2536524). All antibody dilutions were made in TBS containing 15% normal goat serum (Millipore, Burlington, MA, USA) and 0.2% Triton X-100 (Sigma). Finally, sections were rinsed 3 times in TBS for 15 min each and in distilled water for 20 min, allowed to dry for 30 min at 37 °C, and mounted in MOWIOL^®^ 4-88 (Calbiochem). Sections from control and treated animals were always processed in parallel for each antibody staining, and the same antibody solutions were used for sections of control and treated animals in each experiment. Controls omitting the primary antibodies led to no staining at all.

### 2.9. Imaging and Cell Counting of Whole-Mounted Larvae and Spinal Cord Sections

After anti-serotonin immunofluorescence experiments in whole-mounted 4 dpf larvae, confocal photomicrographs were taken at the level of the caudal fin using a Stellaris 8 confocal laser microscope (Leica Microsystems, Wetzlar, Germany) with a 20× objective. For the quantification of serotonergic neurons, the total number of serotonin-immunoreactive interneurons located at the level of the caudal fin (4 spinal cord segments) was quantified manually by going through the stack of confocal optical sections. The experimenter was blinded during the quantifications.

After pH3 or acetylated alpha-tubulin immunofluorescence experiments and TUNEL labelling in cryostat transverse sections, confocal photomicrographs were also taken. Labelling of TUNEL, alpha-tubulin, and pH3 was performed on sections because positive cells for these markers are present in multiple tissues, not only in the spinal cord. Using sections facilitated the reliable identification and quantification of positive cells, specifically within the spinal cord. We quantified the number of pH3+ cells, TUNEL+ nuclei, or the intensity of anti-acetylated tubulin fluorescence (mean grey value) in 1 out of each 3 consecutive spinal cord transverse sections, starting from the caudal end of the spinal cord and moving rostrally, using the Fiji software (version 2.16.0) [17]. Nine sections were quantified in each animal, and then the mean number of labelled cells per section or the mean fluorescence intensity of the spinal cord per section was calculated for each animal. For the calculation of fluorescence intensity of each spinal cord section, the mean intensity of 3 background readings of each image was subtracted from the mean grey value of fluorescence labelling of the spinal cord.

After cell quantifications, the figures were prepared with Adobe Photoshop 2025 (Adobe), with minor adjustments in brightness and contrast of the confocal images.

### 2.10. Statistical Analysis

Each experiment with locomotor analyses or cell/fluorescence quantifications was carried out in a minimum of 2 different clutches of animals (biological replicates) for each different drug treatment, and a minimum of 10 animals were included in each experimental group. Each dot in the graphs represents one animal, and *n* numbers for each experimental group are indicated in the figures or in the Supplementary Files. Statistical analyses were performed with Prism 9 (GraphPad software, La Jolla, CA, USA). Normality of the data was determined with the D’Agostino–Pearson test. For groups with a low *n* number in the first step of the drug screen, we used the Shapiro–Wilk normality test. To determine statistically significant differences (*p* ≤ 0.05) between two groups of normally distributed data, we used an unpaired (Student’s) *t*-test (two-tailed). To determine statistically significant differences between two groups of non-normally distributed data, we used a Mann–Whitney U test (two-tailed). Exact *p*-values are given in the Supplementary Files or in the graphs in the figures.

## 3. Results and Discussion

### 3.1. An Unbiased Drug Screen Reveals That a SIRT2 Inhibitor Reduces the Number of Serotonergic Cells in the Developing Spinal Cord

As indicated earlier (see the Section 1), we first expanded the screen of the LOPAC^®^ Library following the two-step drug-screening protocol developed by González-Llera et al. [8]. In the primary screen, the effect of each drug from the LOPAC^®^ Library was tested (at 10 µM) in 10 larvae. In the secondary screen, hits from the primary screen (drugs that significantly changed the number of serotonergic spinal cord interneurons as compared to DMSO controls) were further tested in groups of 20 larvae.

We screened 80 compounds of the LOPAC^®^ Library (rack 1) in the primary screen (Supplementary File S1). Five of these drugs killed all larvae (Supplementary File S1). Of the remaining 75 drugs, 1 molecule (4-Aminobenzamidine dihydrochloride) led to a minor increase in the number of serotonergic cells, and 30 drugs reduced the number of serotonin-immunoreactive neurons at 4 dpf as compared to DMSO controls (Supplementary File S1). In the secondary screen, with a larger group of animals (*n* = 20), we confirmed that 10 of the 30 compounds significantly reduced the number of spinal cord serotonergic cells (Table 1 and Supplementary File S2). We did not observe a significant change in the number of serotonergic neurons in animals treated with 4-Aminobenzamidine dihydrochloride in the secondary screen (Supplementary File S2).

**Figure 1:**
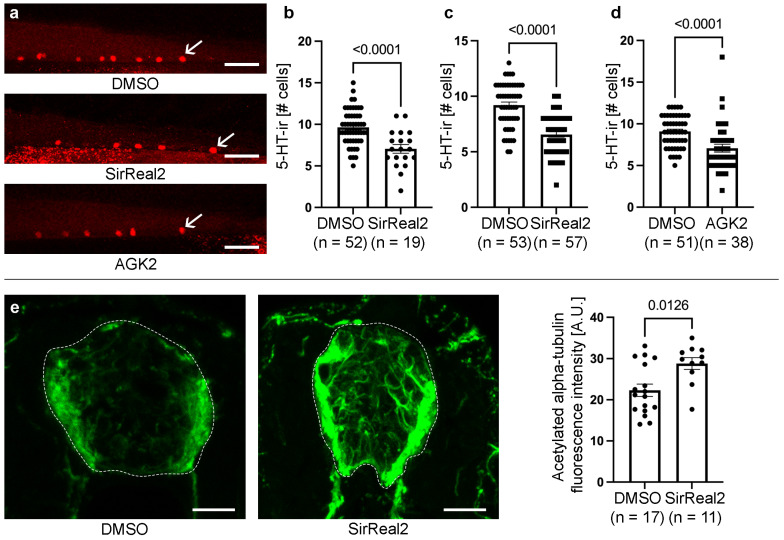
Inhibition of SIRT2 reduces the generation of serotonergic cells in the developing spinal cord. (**a**) Representative photomicrographs of 4 dpf zebrafish showing the reduction in serotonin-immunoreactive cells (arrows) in the caudal spinal cord of SirReal2- or AGK2-treated animals as compared to DMSO controls. (**b**) Graph showing the reduction in the number of serotonin-immunoreactive cells observed in SirReal2-treated animals in the secondary drug screen. (**c**) Graph showing the reduction in the number of serotonin-immunoreactive cells observed in SirReal2-treated animals (newly purchased; not from the drug library) as compared to DMSO controls. (**d**) Graph showing the reduction in the number of serotonin-immunoreactive cells observed in AGK2-treated animals as compared to DMSO controls. (**e**) The SirReal2 treatment increases the intensity of acetylated alpha-tubulin immunofluorescence in the spinal cord of 3 dpf zebrafish. Photomicrographs show acetylated alpha-tubulin immunofluorescence in cryostat spinal cord sections (dotted lines) of DMSO controls and SirReal2-treated 3 dpf larvae. Dorsal is at the top in all photomicrographs. Scale bars: (**a**) 25 µm; (**e**) 15 µm.

Of the 10 hits identified after the two-step drug screen, 2 drugs caused a highly significant (*p* < 0.0001) reduction in the number of serotonergic neurons (Table 1): SirReal2 (Figure 1a,b and Supplementary File S2) and reserpine (Supplementary File S2). SirReal2 is a potent and selective inhibitor of SIRT2 that has an IC_50_ value of 140 nM and is >1000-fold selective for SIRT2 over SIRT1 and SIRT3. Reserpine is a drug known to inhibit the vesicular uptake of catecholamines and serotonin [18]. This could suggest that the drastic reduction in serotonergic neurons in these animals could be related to a loss of serotonin immunoreactivity and not to a true loss of serotonergic neurons in reserpine-treated animals. Thus, for subsequent analyses, we focused on the analysis of the role of SIRT2 in promoting spinal cord neurogenesis.

### 3.2. Pharmacological Experiments Confirm That SIRT2 Activity Regulates Spinal Cord Neurogenesis

To validate the drug screen results, we first repeated the SirReal2 treatment (newly purchased, not from the library) in 2 dpf zebrafish. At 4 dpf, larvae treated with SirReal2 (at 10 µM) showed a significant reduction in the generation of spinal cord serotonergic neurons (Figure 1c). Then, to further confirm that SIRT2 activity promotes the generation of serotonergic neurons in the developing spinal cord, we used a second SIRT2 inhibitor, AGK2. AGK2 is a cell-permeable quinoline compound that targets the nicotinamide-binding site of SIRT2, acting as a reversible selective inhibitor (IC_50_ = 3.5 µM) with little activity against SIRT1 or SIRT3 (IC_50_ > 50 µM). As expected, the AGK2 treatment from 2 to 4 dpf also caused a significant reduction in the generation of spinal cord serotonergic neurons (Figure 1a,d).

Previous work showed that *sirt2−/−* zebrafish adult mutants present elevated levels of tubulin acetylation [19]. To confirm that the SIRT2 inhibitor affects its deacetylase activity in the larval CNS, we analysed the intensity of acetylated alpha-tubulin immunofluorescence in the spinal cord of control and treated larvae. SirReal2-treated 3 dpf animals showed a significant increase in acetylated alpha-tubulin immunofluorescence in the spinal cord as compared to DMSO controls (Figure 1e). Thus, SIRT2 inhibitors applied in the water can reach the larval CNS and reduce the deacetylase activity of SIRT2 in the spinal cord.

These findings support the drug screen results and highlight the role of the deacetylase activity of SIRT2 in promoting the generation of serotonergic neurons. As indicated, only a few previous in vivo studies revealed a role for SIRT2 in regulating neurogenesis in the brain [13,14,15]. Szego et al. [13] showed that SIRT2 knock-out 2-month-old mice present reduced numbers of dopaminergic neurons in the substantia nigra. More recently, decreased levels of SIRT2 in mice models of depression have been linked to reduced neurogenesis [14,15]. Interestingly, SIRT2 overexpression by using oligodendrocyte-derived exosomes was able to enhance neurogenesis in mice with chronic, unpredictable, mild-stress-induced depression [15]. Thus, our study reveals a possible evolutionarily conserved role of SIRT2 in promoting neurogenesis in vertebrates, and reveals for the first time that its deacetylase activity promotes neurogenesis in the spinal cord.

### 3.3. Inhibition of Neurogenesis with SirReal2 Leads to Locomotor Deficits in 4 dpf Larvae

Serotonergic signalling from intrinsic serotonergic spinal cord neurons regulates locomotion by reducing spinally produced motor-bursting in zebrafish [20]. Consequently, previous use of non-steroidal anti-inflammatory drugs, which also reduced the generation of serotonergic neurons in the developing zebrafish spinal cord, led to locomotor deficits in 4 dpf larvae [8]. Thus, it is of interest to test whether the reduction in serotonergic neurons in SirReal2-treated animals is also associated with locomotor deficits.

**Figure 2:**
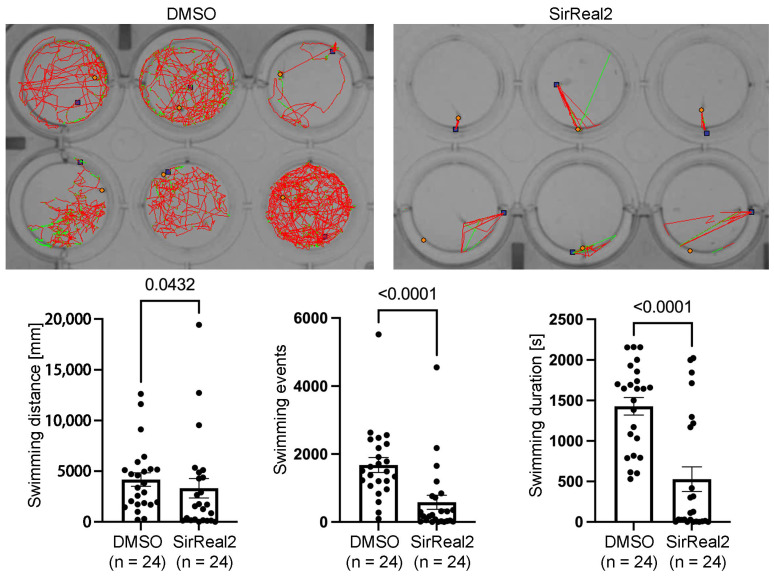
Inhibition of SIRT2 reduces locomotor activity in 4 dpf zebrafish larvae. Images show representative swimming tracks of 6 DMSO controls and 6 SirReal2-treated larvae (the blue squares indicate the starting position, the orange square indicate the end position, red lines indicate long swimming events and green lines indicate short swimming events). Graphs show significant differences in swimming distance, number of swimming events, and duration of swimming in DMSO controls vs. SirReal2-treated animals.

Indeed, locomotor activity was significantly reduced in the SirReal2-treated animals as compared to the DMSO controls (Figure 2). This reduction was evident across all behavioural metrics evaluated: total swimming distance, number of locomotor events, and total duration of activity (Figure 2). The decrease in swimming distance suggests a lower overall displacement, while the reductions in both the event count and duration of the swimming events indicate fewer and shorter activity bouts. Taken together, these results reflect a global attenuation of spontaneous motor behaviour associated to the reduction in serotonergic spinal cord neurons. Whether the generation of other cell types is affected by the SirReal2 treatment should be assessed in future studies. For example, in 2 to 4 dpf larvae, pMN progenitors switch from motor neuron generation to oligodendrocyte generation [21,22,23]. Interestingly, in mammals, SIRT2 is known to be crucial for myelination and is expressed in oligodendrocytes, where its expression is upregulated during oligodendrocyte differentiation [24,25,26,27] (see [11]). Thus, future studies should analyse the possible role of SIRT2 in the generation/differentiation of other neuronal or glial (e.g., oligodendrocytes) cells in the spinal cord, which could also contribute to the observed locomotor deficits.

### 3.4. SIRT2 Promotes Spinal Cord Neurogenesis by Regulating the Mitotic Activity of Progenitor Cells

Analysis of publicly available single-cell RNAseq data reveals *sirt2* transcript expression (ZFIN gene: ZDB-GENE-030131-1028; Ensembl gene: ENSDARG00000011488) in the developing zebrafish spinal cord (“spinal cord/glia” cluster of the Daniocell Atlas; see Supplementary Figure S1a; https://daniocell.nichd.nih.gov, URL accessed on 15 August 2025; [28,29]), including the “radial glia”, “medial floor plate”, “lateral floor plate”, and “spinal cord precursor” subclusters within the major “spinal cord/glia” cluster (see Supplementary Figure S1a). Interestingly, the expression of *sirt2* shows one of the highest correlations of gene expression in this cluster with the *fabp7a* (Supplementary Figure S1b; positive correlation with *r* = 0.122; top nine genes with a positive correlation with *sirt2*), which is a marker of radial glial progenitor cells in the zebrafish CNS [30]. Moreover, anti-SIRT2 immunofluorescence experiments in transverse sections confirmed widespread Sirt2 protein expression in the spinal cord of 2 dpf animals, including the ependymal region, where radial glia progenitors are located (Supplementary Figure S1c). Thus, expression data suggest that SIRT2 could be acting in spinal cord progenitor/precursor cells to promote the generation of serotonergic interneurons in developing animals.

**Figure 3:**
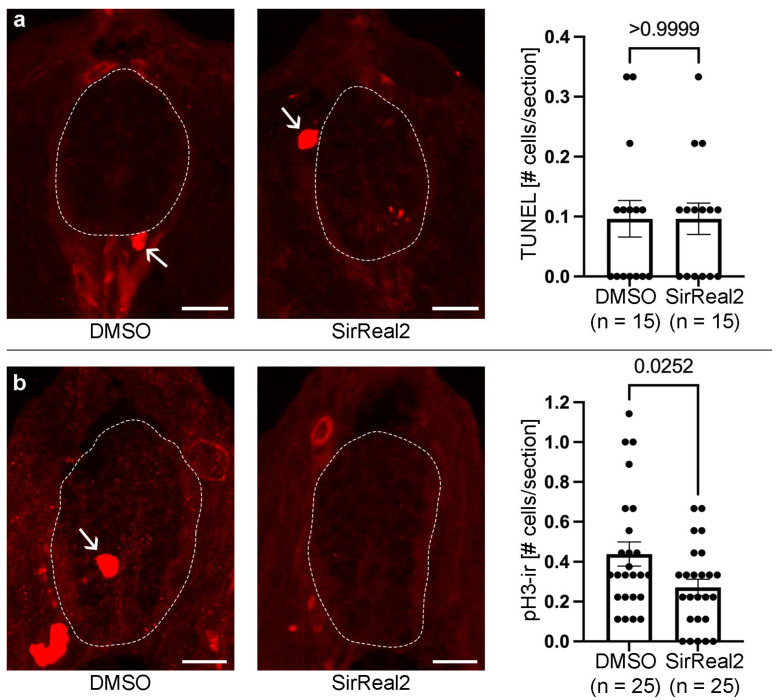
Inhibition of SIRT2 decreases cell proliferation in the spinal cord (dotted lines) of 3 dpf animals. (**a**) Representative photomicrographs of TUNEL labelling in spinal cord sections and graph showing that SirReal2-treated animals do not present changes in the number of apoptotic spinal cord cells (TUNEL+). Note the presence of TUNEL+ cells in neighbouring tissues (arrows) that serve as a positive control for the TUNEL labelling. (**b**) Representative photomicrographs of pH3 immunofluorescence in spinal cord sections and graph showing a reduction in pH3+ (mitotic) cells (arrows) in SirReal2-treated animals. Dorsal is at the top in all photomicrographs. Scale bars: (**a**,**b**), 10 µm.

To determine the cellular mechanisms by which SIRT2 promotes neurogenesis, we analysed the effect of the SirReal2 treatment on apoptotic cell death and mitotic activity in the spinal cord of 3 dpf zebrafish. We did not observe a significant difference in the (very low) number of TUNEL+ (apoptotic) cells in the spinal cord between DMSO controls and SirReal2-treated 3 dpf zebrafish (Figure 3a). This indicates the following: (1) SIRT2 inhibition does not reduce neurogenesis by causing an increase in apoptotic cell death; (2) SIRT2 does not promote neurogenesis by promoting the survival of progenitor cells or differentiating/differentiated neurons. However, we found a significant decrease in mitotic activity (reduced numbers of pH3+ cells) in the spinal cord of SirReal2-treated animals as compared to DMSO controls (Figure 3b). These results indicate that SIRT2 promotes neurogenesis, at least in part, by promoting mitotic activity in progenitor cells.

The role of SIRT2 in regulating mitotic activity was one of the earliest SIRT2 functional discoveries. SIRT2 accumulates in the nucleus during mitosis to maintain normal mitotic activity (see [11]). In the nervous system, olfactory bulbectomised mice show reduced SIRT2 expression, which is associated with reduced cell proliferation as revealed by decreased BrdU labelling in the hippocampus [14]. Here, the expression data and pharmacological manipulations suggest that SIRT2 promotes mitotic activity in progenitor cells of the developing spinal cord in zebrafish.

Regarding the molecular mechanisms through which SIRT2 regulates mitotic activity, we observed that SIRT2 inhibition in the zebrafish spinal cord led to increased levels of alpha-tubulin acetylation (see above). Alpha-tubulin is a well-established deacetylation substrate of SIRT2 [31], and its mitotic acetylation is crucial for proper mitotic spindle microtubule function [32,33,34,35]. Notably, disruption of several regulators of the microtubule deacetylase HDAC6 also results in mitotic defects associated with aberrant microtubule acetylation [34,36,37,38]. Our observations suggest that SIRT2 may control mitosis in spinal cord progenitor cells through its deacetylase activity on alpha-tubulin. Future studies should aim to test this hypothesis to delineate the molecular pathways by which SIRT2 regulates mitotic activity. Such mechanisms could involve alpha-tubulin deacetylation—as suggested by our data—and/or the deacetylation of other mitosis-related substrates, including the histone methyltransferase SMC1A, PR-Set7, CDH1, GRASP55, or CDC20 [39,40] (see [11]).

## 4. Conclusions

Using our unbiased drug-screening protocol in zebrafish, we identified a role for the deacetylase SIRT2 in promoting neurogenesis in the developing spinal cord. Expression data and pharmacological manipulations indicate that SIRT2 enhances the generation of serotonergic interneurons, at least in part, by regulating the mitotic activity of progenitor cells, likely through its deacetylation of alpha-tubulin. Future studies should analyse whether the activity of other progenitor cells (e.g., pMN progenitors) is also regulated by SIRT2.

Our findings not only pinpoint a previously underappreciated regulator of vertebrate neurogenesis but could also highlight SIRT2 as a potential therapeutic target to stimulate cell proliferation and neurogenesis following traumatic or degenerative damage to the CNS.

## Figures and Tables

**Table 1:** Hits reducing the generation of serotonergic neurons in the spinal cord identified after the 2-step drug-screening protocol.

Drug	*p*-Value	Application
SirReal2	<0.0001	Potent and selective inhibitor of SIRT2
Reserpine	<0.0001	Inhibits vesicular uptake of catecholamines and serotonin
YM 976	0.0001	Phosphodiesterase type IV inhibitor
Atreleuton	0.0002	Reversible 5-lipoxygenase inhibitor
Arcaine sulfate	0.0011	N-methyl-D-aspartate receptor effector/antagonist
L-732,138	0.0029	Competitive substance P receptor antagonist
Acetylthiocholine chloride	0.0029	Acetylcholinesterase substrate and nicotinic acetylcholine receptor agonist
Actinonin	0.0135	Inhibitory action against peptide deformylase
UNC0379 trifluoroacetate salt	0.0156	First substrate-competitive inhibitor of the lysine methyltransferase SETD8
Azelaic acid	0.0215	Antibacterial, anti-keratinising, antimelanogenic, antioxidant, and anti-inflammatory agent

## Data Availability

Raw imaging data are available from the authors upon reasonable request.

## Supplementary Materials

The following supporting information can be downloaded at https://www.mdpi.com/article/10.3390/biom15101359/s1, Supplementary Figure S1: a. Spinal cord/glia cluster of the Daniocell scRNAseq Atlas showing expression of *sirt2* in radial glia, medial floor plate, lateral floor plate, and spinal cord precursors subcluster. b. Table from the Daniocell Atlas showing the top 9 genes showing positively correlated gene expression with *sirt2*. Note that the radial glia marker *fabp7a* is in the top 9 genes with a positive correlation (highlighted in yellow). c. Photomicrograph of a spinal cord section of a 2 dpf larva showing widespread expression of SIRT2, including the ependymal region. Scale bar: 10 µm; Supplementary File S1: Data from the first step of the drug screen; Supplementary File S2: Data from the second step of the drug screen.
