# Supplementary figures and images for: An In Vivo Drug Screen Reveals That Sirtuin 2 Activity Promotes Spinal Cord Neurogenesis in Developing Zebrafish
Source: Biomolecules. 2025 Sep 24;15(10):1359. doi: 10.3390/biom15101359 (PMC12564900; doi:10.3390/biom15101359)

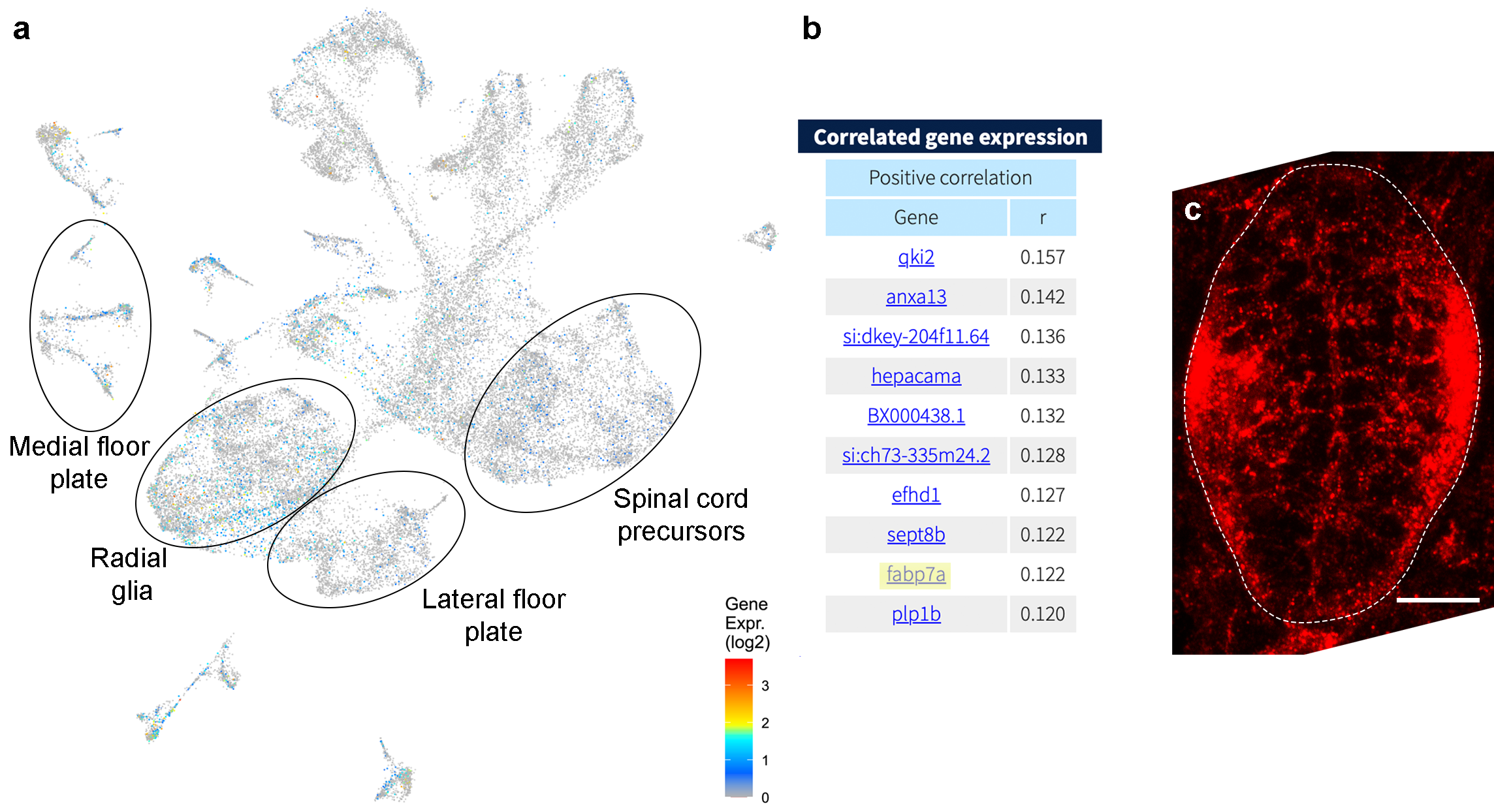

Supplement: Supplementary file 1 [file biomolecules-15-01359-s001.zip › Supplementary Figure S1.tif]
